# Assessment of textbook outcome after lobectomy for early‐stage non‐small cell lung cancer in a Korean institution: A retrospective study

**DOI:** 10.1111/1759-7714.14391

**Published:** 2022-03-20

**Authors:** Woo Sik Yu, Jaeyong Shin, Jung A Son, Joonho Jung, Seokjin Haam

**Affiliations:** ^1^ Department of Thoracic and Cardiovascular Surgery Ajou University School of Medicine Suwon Korea; ^2^ Department of Preventive Medicine Yonsei University College of Medicine Seoul Korea

**Keywords:** carcinoma, healthcare, non‐small cell lung cancer, quality indicators, thoracic surgery

## Abstract

**Background:**

Textbook outcome (TO) has been introduced as a novel composite measure for lung cancer surgery. We investigated TO after lobectomy for early‐stage non‐small cell lung cancer (NSCLC) in a Korean tertiary hospital and its prognostic implications for overall survival and recurrence.

**Methods:**

Between January 2012 and December 2017, 418 consecutive patients who underwent lobectomy for clinical stages I and II NSCLC were identified and retrospectively reviewed. TO was defined as complete resection (negative resection margins and sufficient lymph node dissection), no 30‐day or in‐hospital mortality, no reintervention within 30 days, no readmission to the intensive care unit, no prolonged hospital stay (<14 days), no hospital readmission within 30 days, and no major complications. Propensity score matching analysis was performed to investigate the association between TO, medical costs, and long‐term outcomes.

**Results:**

Of 418 patients, 277 (66.3%) achieved TO. The most common events leading to TO failure were prolonged air leakage (*n* = 54, 12.9%) and prolonged hospital stay (*n* = 53, 12.7%). Male sex (odds ratio [OR] = 2.148, *p* = 0.036) and low diffusing capacity for carbon monoxide (OR = 0.986, *p* = 0.047) were significant risk factors for failed TO in multivariate analysis. In matched cohorts, achieving TO was associated with lower medical costs and better overall survival but not cancer recurrence.

**Conclusions:**

TO is associated with low medical cost and favorable overall survival; thus, surgical teams and hospitals should make efforts to improve the quality of care and achieve TO.

## Graphical abstract

This study analyzed textbook outcome (TO) after lobectomy for early stage non‐small cell lung cancer in a Korean tertiary institution. The rate of achievement of TO and the cause of failure in achieving TO were quite different from the previous reports from western countries (achievement of TO, 66.3%; leading cause of failure, prolonged air leakage [*n* = 54, 12.9%], prolonged hospital stay [*n* = 53, 12.7%]). TO is associated with low medical costs and favorable overall survival; thus, surgical teams and hospitals should make efforts to improve the quality of care and achieve TO.

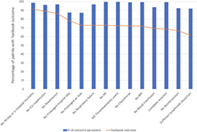

## INTRODUCTION

Lung cancer is the leading cause of cancer‐related death in Korea and worldwide.^1^, ^2^ Non‐small cell lung cancer (NSCLC) accounts for approximately 85% of lung cancers, and surgery is the mainstay of treatment for early‐stage NSCLC.^3^ The total number of lung cancer surgeries performed has increased over time in Korea. However, the number of extensive resections, such as bilobectomy or pneumonectomy, has decreased.^4^, ^5^ This trend may be associated with the increased use of computed tomography (CT), which results in more lung cancer detected in earlier stages, indicating less extensive surgical treatment. Early detection and increased use of surgical treatment may be related to recent improvements in the prognosis of lung cancer in Korea,^6^ and this trend may continue in the future with the increased use of low‐dose chest CT as a lung cancer screening program.^7^

The quality of surgical care may have a great impact on the short‐ and long‐term outcomes of lung cancer surgery.^8^ In addition, quality measurements can help patients choose hospitals and lead hospitals and their health care workers to improve their quality of surgical care. To define the quality of surgical care, various metrics have been proposed.^9^, ^10^ However, quality should be a multidimensional construct that cannot be measured using a single metric.^10^ Textbook outcome (TO) has been introduced as a novel composite measure in various oncological surgical fields, such as colon, esophagogastric, and pancreatic cancer surgeries.^11^, ^12^, ^13^ TO defines such composite measurements by accounting for multiple postoperative endpoints that may reflect the ideal or “textbook” postoperative outcomes.^14^

Recently, TO was defined and analyzed in lung cancer surgery using nationwide cohorts, including the Dutch Lung Cancer Audit‐Surgery (DLAS‐S) of the Netherlands and the National Cancer Database (NCBD) of the United States.^15^, ^16^ TO was achieved in about 26% of cases in both series, and insufficient lymph node (LN) assessment was the most frequent cause of failure to achieve TO (DLAS‐S, 55%; NCBD, 59.2%). Kulshrestha et al. also showed an association between the achievement of TO and improved overall survival (OS) in their analysis of the NCBD, but they could not assess cancer‐specific outcomes because of the lack of data.^15^ Intercontinental variation in lung cancer surgery has been suggested, especially in LN examinations, but TO has never been analyzed in Asia.^17^ In the present study, we investigated TO after lobectomy for early‐stage NSCLC in a Korean tertiary hospital and its prognostic implications for OS and recurrence.

## METHODS

### Patients

In reviewing electronic medical records, we identified patients with NSCLC who underwent surgical treatment between January 2012 and December 2017. Among them, we identified patients who underwent lobectomy for clinical stage I and II NSCLC. Patients who underwent sublobar resection, bilobectomy, or pneumonectomy were excluded because the ideal postoperative course (TO) may have differed according to the extent of lung resection. We also excluded patients who received neoadjuvant treatment, those with pleural seeding incidentally detected intraoperatively, and those with adenocarcinoma in situ or microinvasive adenocarcinoma because these may have influenced the operation and extent of LN dissection.

### Data collection

Patient demographic and clinical characteristics included age, sex, smoking status, Charlson comorbidity index (CCI),^18^ pulmonary function test, tumor histology, harvested LN, clinical and pathological stage, operation approach, and medical cost. Staging was determined by the eighth edition of the tumor‐node‐metastasis (TNM) classification.^19^ Medical cost was calculated as the total cost during index hospitalization and readmission within 30 days. Follow‐up for this study was completed in August 2021.

### Definition of textbook outcome

We followed the definition for TO from the Dutch Lung Cancer Audit.^16^ TO was achieved when all the following desired outcomes were satisfied: negative resection margin and sufficient resection margin, no 30‐day or in‐hospital mortality, no reintervention (reoperation, bronchoscopy for atelectasis, and percutaneous drainage) within 30 days after the primary operation, no readmission to the intensive care unit (ICU) or prolonged ICU stay due to complications, no prolonged hospital stay (<14 days), no hospital readmission within 30 days, no major complications including prolonged air leakage (≥5 days), respiratory failure (acute respiratory distress syndrome and pulmonary edema), myocardial infarction, thromboembolic complications, chylothorax, empyema and/or bronchopleural fistula (BPF), and blood transfusion. Sufficient LN dissection followed the Union for International Cancer Control (UICC) recommendation. It was defined as the dissection or sampling of at least six LNs: three removed from intrapulmonary and/or hilar stations, and three removed from mediastinal stations, one of which must be the subcarinal station.^20^

### Statistical analyses

Continuous variables were compared using a Student's *t*‐test. Categorical variables were compared using the chi‐square test or Fisher's exact test. Risk factors for failed TO were analyzed using a binary logistic regression test. We constructed a multivariable model with clinically relevant variables (full model) and another model with backward stepwise selection (step wise model). Propensity score‐matched analysis was performed to overcome the differences in preoperative variables. Propensity scores were calculated using a logistic regression model that included the following variables: age, sex, smoking status, CCI, pulmonary function test results (forced expiratory volume in 1 second and diffusing capacity for carbon monoxide [DLCO]), histology, tumor size, and clinical stage. Propensity scores were then matched to obtain pairs of matched patients in a 1:1 manner, using nearest matching without replacement. The balance of covariates between the failed TO group (those who failed TO) and achieved TO group (those who achieved TO) was assessed using standardized mean differences, with adequacy considered to be <0.2.

OS was calculated using the Kaplan–Meier method and compared using the log‐rank test. The cumulative incidence of recurrence with death as a competing risk factor was compared using the Fine and Gray method. Statistical significance was set at p ≤ 0.05. Statistical analyses were performed using R version 4.1.0 (R Foundation for Statistical Computing).

## RESULTS

### Baseline characteristics

**FIGURE 1:**
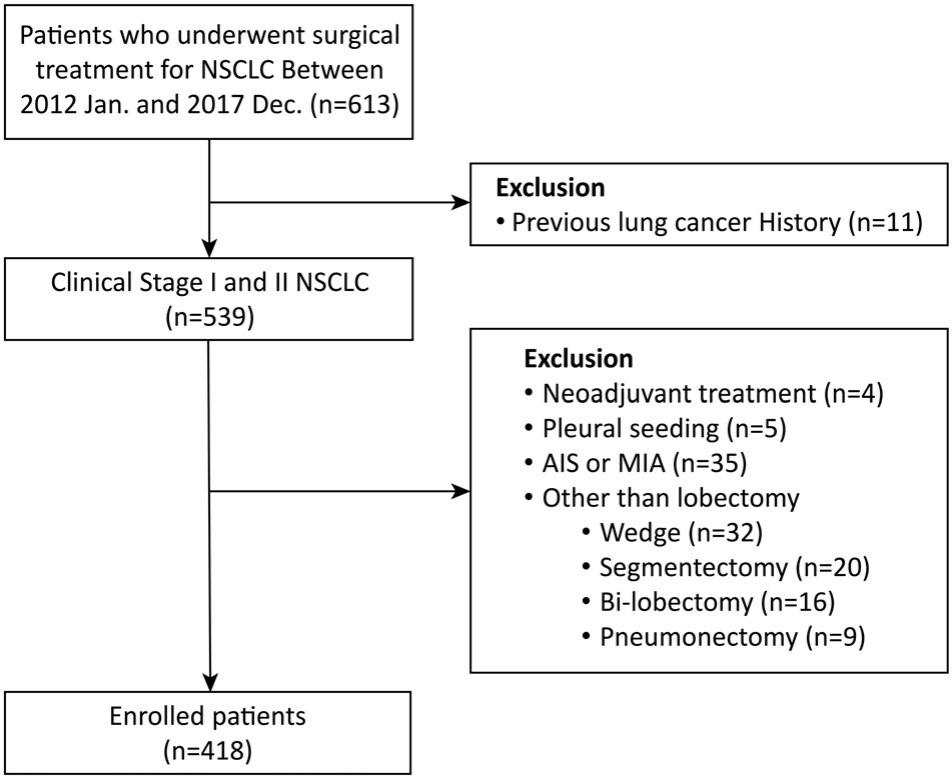
Diagram of the study cohort selection process. AIS, adenocarcinoma in situ; MIA, microinvasive adenocarcinoma; NSCLC, non‐small cell lung cancer

A total of 418 patients were enrolled in this study (Figure 1). The demographic and medical characteristics are presented in Table 1. Of 418 patients, 277 (66.3%) achieved TO. Compared to the failed TO group, the achieved TO group showed a lower proportion of men, less smoking history, lower CCI, and higher DLCO. The achieved TO group was more likely to have adenocarcinomas and smaller tumors than the failed TO group. More LNs were harvested during surgery in the achieved TO group than in the failed TO group.

**TABLE 1:** Baseline characteristics of the achieved and failed textbook outcome groups

Variable	All patients (*N* = 418)	Achieved TO (*N* = 277)	Failed TO (*N* = 141)	*p*‐value
Age, years, mean ± SD	63.6 ± 10.0	63.0 ± 9.9	64.9 ± 10.1	0.060
Sex				<0.001
Male	273 (65.3%)	163 (58.8%)	110 (78.0%)	
Female	145 (34.7%)	114 (41.2%)	31 (22.0%)	
Smoking				<0.001
Non‐smoker	163 (39.0%)	126 (45.5%)	37 (26.2%)	
Ex‐smoker	128 (30.6%)	82 (29.6%)	46 (32.6%)	
Current smoker	127 (30.4%)	69 (24.9%)	58 (41.1%)	
CCI				0.018
0	197 (47.1%)	143 (51.6%)	54 (38.3%)	
1	94 (22.5%)	61 (22.0%)	33 (23.4%)	
>2	127 (30.4%)	73 (26.4%)	54 (38.3%)	
FEV1, % predicted	93.0 ± 13.8	93.5 ± 12.9	91.9 ± 15.3	0.271
DLCO, % predicted	77.0 ± 20.0	80.0 ± 19.3	71.0 ± 20.1	<0.001
Approach				0.009
VATS	277 (66.3%)	196 (70.8%)	81 (57.4%)	
Thoracotomy	141 (33.7%)	81 (29.2%)	60 (42.6%)	
Histology				<0.001
Adeno	286 (68.4%)	208 (75.1%)	78 (55.3%)	
Sq	97 (23.2%)	47 (17.0%)	50 (35.5%)	
Other NSCLC	35 (8.4%)	22 (7.9%)	13 (9.2%)	
Tumor size, cm	3.1 ± 1.7	2.9 ± 1.5	3.4 ± 2.0	0.014
Harvested LNs, *n*	24.0 ± 11.5	25.0 ± 11.0	22.0 ± 12.1	0.010
Clinical stage				0.221
IA	151 (34.1%)	96 (34.7%)	55 (39.0%)	
IB	193 (43.2%)	137 (49.5%)	56 (39.7%)	
IIA	10 (2.4%)	5 (1.8%)	5 (3.5%)	
IIB	64 (15.3%)	39 (14.1%)	25 (17.7%)	
Pathological stage				0.271
IA	129 (30.9%)	88 (31.8%)	41 (29.1%)	
IB	145 (34.7%)	104 (37.5%)	41 (29.1%)	
IIA	25 (6.0%)	14 (5.1%)	11 (7.8%)	
IIB	59 (14.1%)	33 (11.9%)	26 (18.4%)	
IIIA	52 (12.4%)	33 (11.9%)	19 (13.5%)	
IIIB	8 (1.9%)	5 (1.8%)	3 (2.1%)	

Abbreviations: Adeno, adenocarcinoma; CCI, Charlson comorbidity index; DLCO, diffusing capacity for carbon monoxide; FEV1, forced expiratory volume in 1 s; LN, lymph node; SD, standard deviation; TO, textbook outcome; VATS, video‐assisted thoracoscopic surgery; NSCLC, non‐small cell lung cancer; Sq, squamous cell carcinoma.

### Prevalence of events leading to textbook outcome failure

**FIGURE 2:**
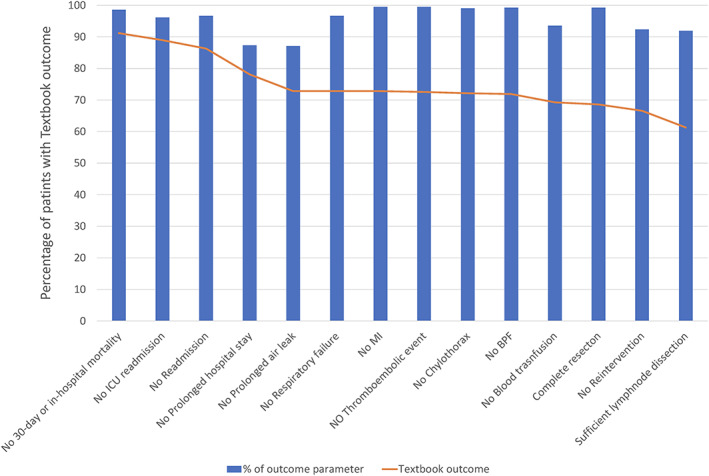
Diagram representing TO as composite measure of outcome parameters. TO, textbook outcome

**FIGURE 3:**
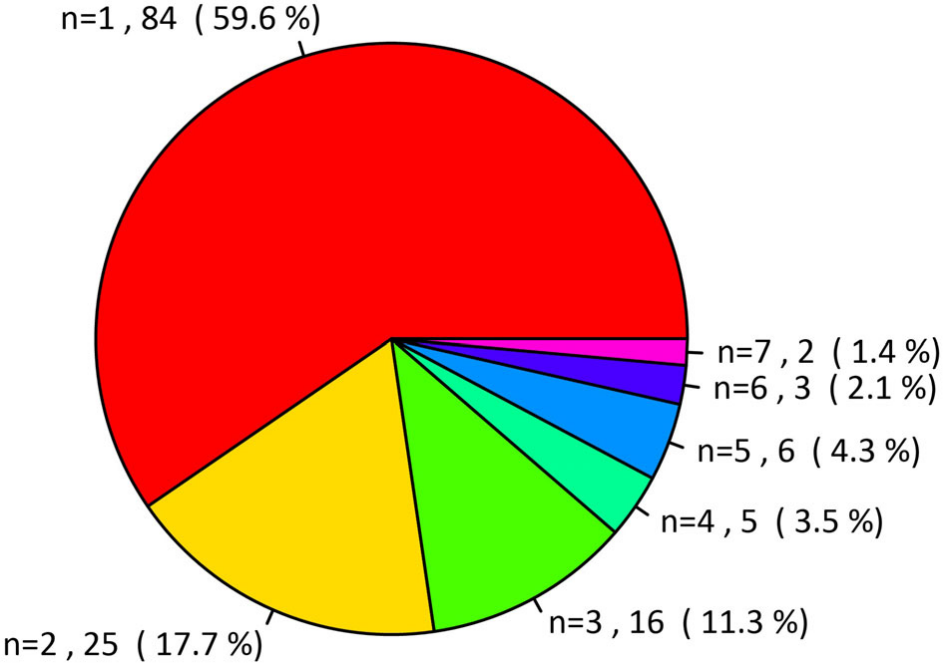
Number of complications defining TO failure among patients who failed TO achieve TO. TO, textbook outcome

The most common events leading to TO failure were prolonged air leakage (*n* = 54, 12.9%) and prolonged hospital stay (*n* = 53, 12.7%). The patients with prolonged air leakage had significantly longer hospital stays compared with patients without prolonged air leakage (17.1 ± 11.4 vs. 8.4 ± 8.2, *p* < 0.001, respectively) and 30 patients (55.6%) with prolonged air leakage also had a prolonged hospital stay. Of 54 patients with prolonged air leakage, 39 (72.2%) patients received bedside pleurodesis, and two (3.7%) patients underwent reoperation with general anesthesia. The least common events were myocardial infarction (*n* = 2, 0.5%) and empyema/BPF (*n* = 3, 0.7%) (Figure 2 and Table 2). Among patients with failed TO, 84 (59.6%) had a single complication (Figure 3).

**TABLE 2:** Prevalence of events leading to textbook outcome failure

Variable	Total patients (*n* = 418)
30‐day or in‐hospital mortality	6 (1.4%)
ICU readmission or prolonged stay	16 (3.8%)
Readmission with 30 days	14 (3.3%)
Prolonged hospital stay (≥14 days)	53 (12.7%)
Prolonged air leakage (≥5 days)	54 (12.9%)
Respiratory failure	14 (3.3%)
Myocardial infarction	2 (0.5%)
Thromboembolic event	2 (0.5%)
Chylothorax	4 (1.0%)
Empyema or BPF	3 (0.7%)
Blood transfusion	27 (6.5%)
Incomplete resection	3 (0.7%)
Insufficient LN dissection	34 (8.1%)

Abbreviations: BPF, bronchopleural fistula; ICU, intensive care unit; LN, lymph node.

### Risk factors for textbook outcome failure

Risk factors for failed TO according to binary logistic regression analysis are shown in Table 3. In the full model with possible relevant variables and stepwise model with backward selection, male sex and low DLCO were significant risk factors for failed TOs.

**TABLE 3:** Predicted factors for achievement of textbook outcome according to the logistic regression model

Variable	Full model			Stepwise model		
	OR	95% CI	*p*‐value	OR	95% CI	*p*‐value
Age	0.997	0.970–1.025	0.836			
Male (vs. female)	2.148	1.050–4.405	0.036	2.162	1.350–3.526	0.002
Smoking						
Non‐smoker	Reference					
Ex‐smoker	0.711	0.325–1.559	0.391			
Current smoker	1.178	0.551–2.523	0.672			
CCI ≥ 2	1.582	0.985–2.538	0.057	1.501	0.949–2.365	0.081
Preop FEV1, % predicted	0.999	0.982–1.016	0.882			
Preop DLCO, % predicted	0.986	0.972–1.000	0.047	0.982	0.971–0.993	0.002
VATS (vs. open)	0.727	0.451–1.178	0.193	0.650	0.416–1.017	0.058
Histology						
Adeno	Reference					
Sq	1.618	0.919–2.846	0.095			
Other NSCLC	1.141	0.511–2.455	0.741			
Tumor size, cm	1.121	0.963–1.311	0.144			
Clinical stage						
I	Reference					
II	0.699	0.363–1.313	0.273			

Abbreviations: Adeno, adenocarcinoma; CCI, Charlson comorbidity index; CI, confidence interval; DLCO, diffusing capacity for carbon monoxide; FEV1, forced expiratory volume in 1 s; NSCL, non‐small cell lung cancer; OR, odds ratio; Sq, squamous cell carcinoma; VATS, video‐assisted thoracoscopic surgery.

### Propensity‐matched analysis

To investigate the impact of TO achievement on long‐term outcomes, we performed propensity‐matched analysis, which resulted in 128 well‐balanced pairs of patients (Table 4).

**TABLE 4:** Baseline characteristics of the achieved and failed textbook outcome groups in the matched cohort

	Achieved TO (*n* = 128)	Failed TO (*n* = 128)	SMD
Age, years, mean ± SD	63.5 ± 9.6	64.4 ± 10.3	0.096
Male sex	100 (78.1)	99 (77.3)	0.019
Smoking			0.055
Non‐smoker	36 (28.1)	36 (28.1)	
Ex‐smoker	41 (32.0)	44 (34.4)	
Current smoker	51 (39.8)	48 (37.5)	
CCI			0.073
0	50 (39.1)	52 (40.6)	
1	33 (25.8)	29 (22.7)	
≥2	45 (35.2)	47 (36.7)	
Preop FEV1, % predicted	95.52 (13.03)	92.26 (14.67)	0.019
Preop DLCO, % predicted	74.93 (18.20)	72.90 (19.46)	0.108
VATS	79 (61.7)	77 (60.2)	0.032
Histology			0.027
Adeno	75 (58.6)	76 (59.4)	
Sq	40 (31.2)	40 (31.2)	
Other NSCLC	13 (10.2)	12 (9.4)	
Tumor size, cm	3.2 ± 1.5	3.2 ± 1.7	0.013
Clinical stage			0.163
IA	47 (36.7)	50 (39.1)	
IB	57 (44.5)	54 (42.2)	
IIA	5 (3.9)	2 (1.6)	
IIB	19 (14.8)	22 (17.2)	
Pathological stage			0.144
IA	40 (31.2)	38 (29.7)	
IB	41 (32.0)	40 (31.2)	
IIA	11 (8.6)	10 (7.8)	
IIB	16 (12.5)	21 (16.4)	
IIIA	15 (11.7)	16 (12.5)	
IIIB	5 (3.9)	3 (2.3)	

Abbreviations: Adeno, adenocarcinoma; CCI, Charlson comorbidity index; DLCO, diffusing capacity for carbon monoxide; FEV1, forced expiratory volume in 1 s; NSCL, non‐small cell lung cancer; SD, standard deviation; SMD, standardized mean difference; VATS, video‐assisted thoracoscopic surgery; Sq, squamous cell carcinoma.

**FIGURE 4:**
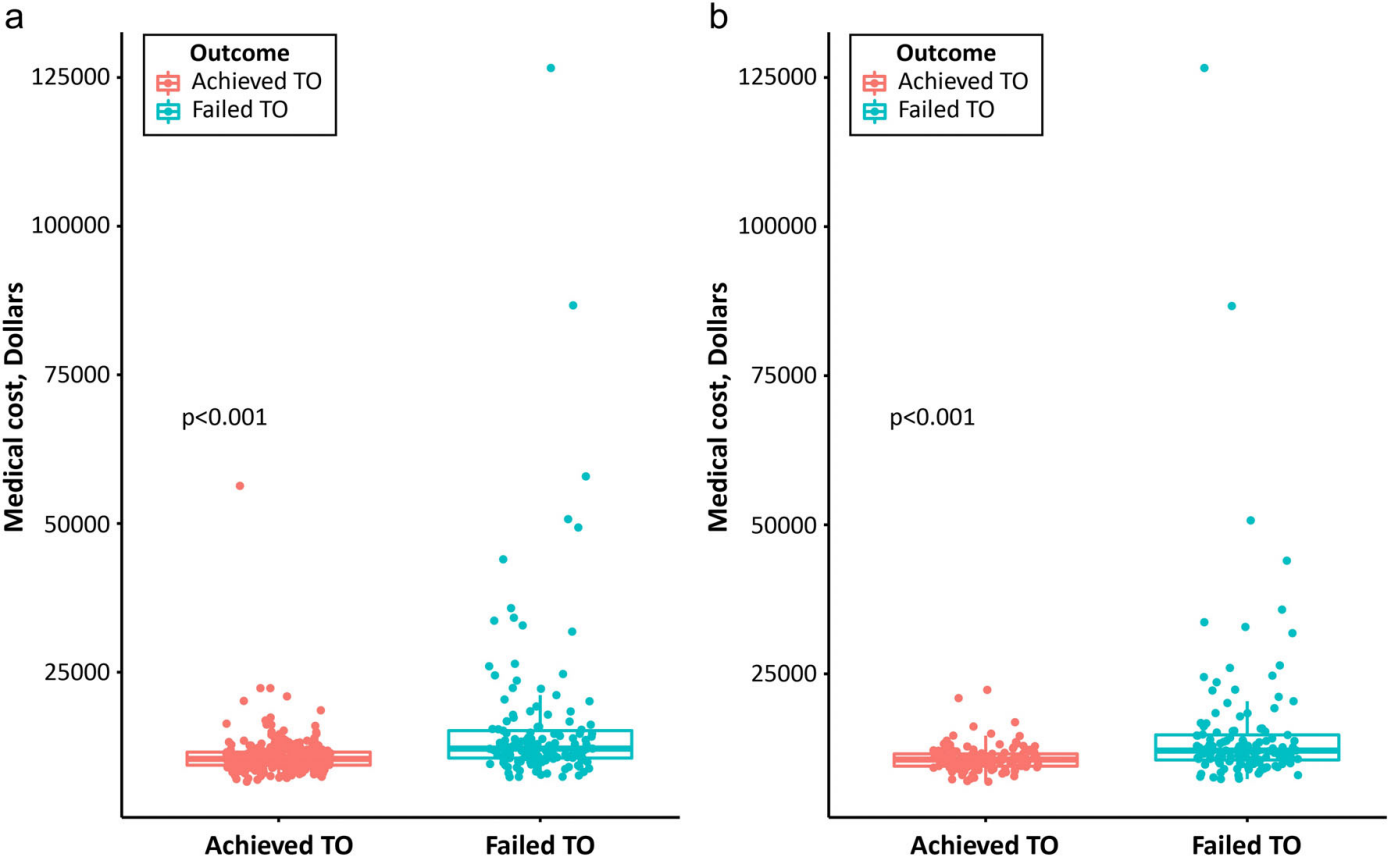
Financial implications of textbook outcome (TO) in unmatched and matched cohorts. (a) Unmatched cohorts. (b) Matched cohort

In both unmatched and matched cohorts, medical costs were 1.5 times higher for failed TO than for achieved TO (unmatched cohorts, $15936.7 ± $13882.8 vs. $10912.1 ± $3574.1, respectively, *p* <0.001; matched cohort, $15471.8 ± $13610.7 vs. $10813.6 ± $2208.8, respectively, *p* <0.001; Figure 4).

**FIGURE 5:**
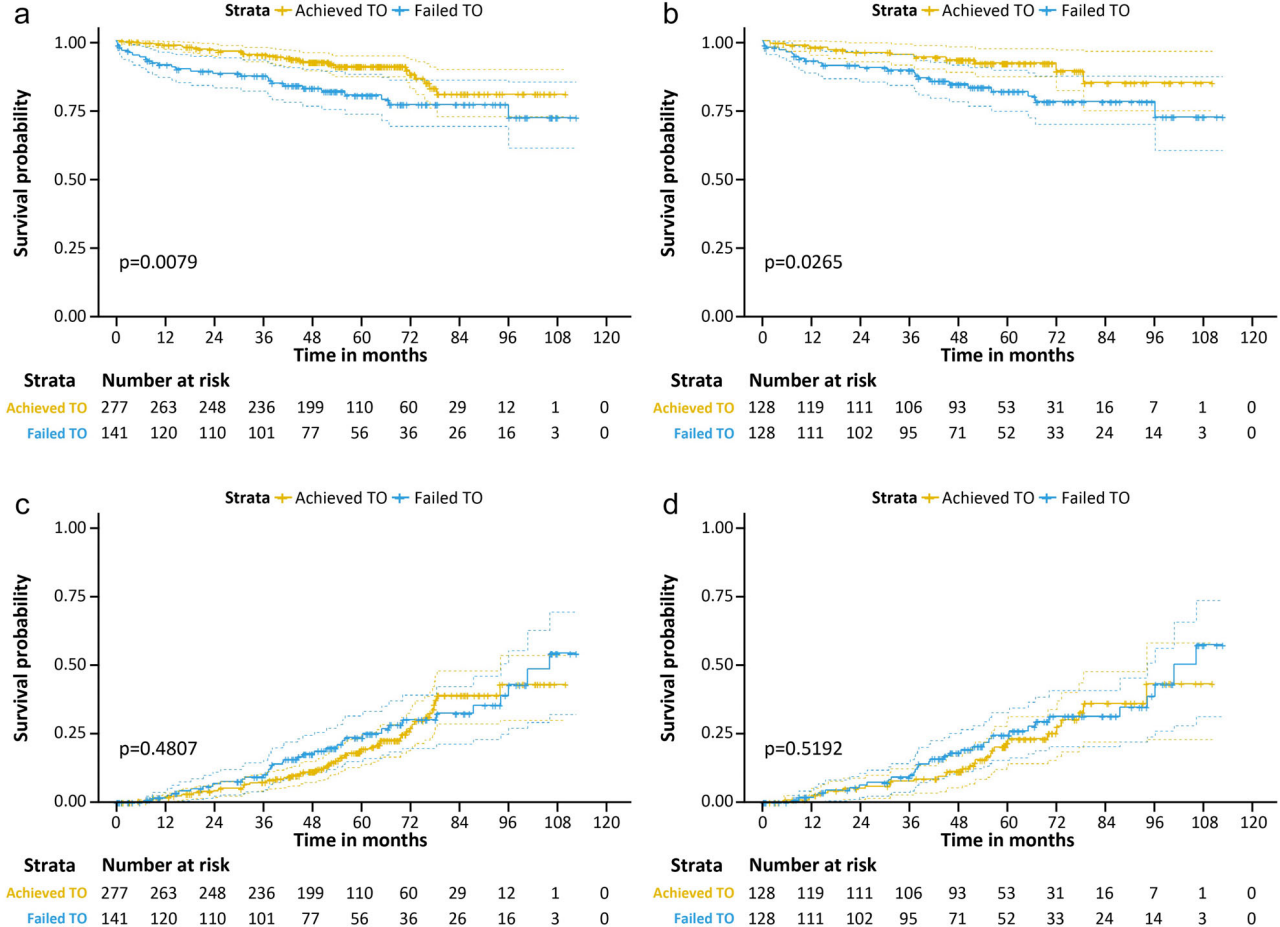
Overall survival in the unadjusted (a) and matched cohorts (b). Cumulative incidence of recurrence in the unadjusted (c) and matched cohorts (d). TO, textbook outcome

In unmatched cohorts, the achieved TO group had better 3‐ and 5‐year OS rates than the failed TO group (95.0% and 90.7% vs. 87.4% and 80.3%, respectively; *p* = 0.018). There was no significant difference in the cumulative incidence of recurrence at 3‐ and 5‐years between the achieved TO and failed TO groups (7.2% and 18.6% vs. 9.1% and 23.5%, respectively; *p* = 0.481). In matched cohorts, the achieved TO group still had better 3‐ and 5‐year OS rates than the failed TO group (95.0% and 92.0% vs. 89.3% and 81.7%, respectively; *p* = 0.027). There was no significant difference in the cumulative incidence of recurrence at 3‐ and 5‐years between the achieved TO and failed TO groups (7.5% and 21.5% vs. 9.0% and 24.4%, respectively; *p* = 0.481) (Figure 5) .

## DISCUSSION

To the best of our knowledge, this is the first study to analyze TO in lung cancer patients in Asia. Of 418 patients enrolled into the study who underwent lobectomy for clinical stage I and II NSCLC, 66.3% of patients achieved TO. Prolonged air leakage and hospital stay were the most frequent causes of TO failure. Male sex and a low DLCO were significant risk factors for failed TO. TO was associated with lower medical costs and favorable OS but not recurrence in the propensity‐matched analysis.

Defining, measuring, and delivering high‐quality surgical care in patients with lung cancer is complex.^10^ Various metrics have been suggested, and none of the single metrics can fully reflect all the multistep processes in lung cancer surgical care.^9^ Postoperative morbidity and mortality are considered important quality metrics. However, the individual event rate and variation of these events are low, which makes them less suitable for detecting hospital variations and measuring quality improvement.^16^

TO represents ideal postoperative outcomes with various components and results in binary outcomes (achieved or failed TO). This is more understandable and informative for patients than a single metric is. Furthermore, variations in TO between hospitals and surgeons may be larger than a single metric and may provide information about their specific improvement potentials. In the present study, 33.7% of patients had a failed TO. Prolonged air leakage and hospital stay were the most frequent causes of failure in 12.9% and 12.7% of cases, respectively and 55.6% of patients with prolonged air leakage also had prolonged hospital stay, whereas the failure rates of other criteria were < 10%. Additionally, 59.6% of the patients with failed TO failed only one of the criteria. This suggests that we have the potential to improve our TO, and efforts need to be made to reduce cases of air leakage at our institution.

The DLAS‐S used the definition of dissection or sampling of a minimum of three mediastinal LNs (including at least one in the subcarinal station) and hilar and intrapulmonary LNs according to the European Society of Thoracic Surgeons guidelines.^21^ Kulshrestha et al. used a threshold of 10 LNs for sufficient LN assessment to be consistent with the Commission on Cancer quality metric in the analysis of NCBD data.^15^ In these studies, the insufficient LN assessment was the most frequent cause of failure to achieve TO.^15^, ^16^ In the present study, we used the Union for International Cancer Control recommendation for criteria of sufficient LN dissection, which was defined as at least three LNs removed from the N1 stations and three LNs removed from the N2 stations. Despite the stricter definition for sufficient LN dissection, the failure of sufficient LN dissection was only 8.1%.

The International Association for the Study of Lung Cancer Staging and Prognostic Factors Committee demonstrated significant differences in the survival of patients with pN0, pN1, and pN2 NSCLCs in the international database.^22^, ^23^ Asian patients (mostly from South Korea and Japan) with pN0 disease had a 5‐year survival rate of 79% compared with 5‐year survival rates of 67% for Americans, 58% for Australians, and 54% for Europeans. The disparities in survival of patients with pN2 were much lower (39, 42, 33, and 22%, respectively). One of the hypotheses for these survival differences is the intercontinental variation in lung cancer surgery, especially in the thoroughness of the LN examination.^17^ In the present study, the mean number of resected LNs was 24.0 ± 11.5, which is consistent with that in previous reports from other Korean tertiary centers.^24^, ^25^, ^26^, ^27^ Approximately 80% of lung cancer surgeries are performed in tertiary centers in Korea,^5^ so the failure rate of sufficient LN dissection in Korea might also be quite low compared with that in previous reports from the DLAS‐S and NCBD. However, to confirm this postulation, a nationwide database including details of LN evaluations needs to be established in Korea.

In the current study, male sex and low DLCO were risk factors for failed TO. Additionally, in previous reports, age, comorbidities, lung function, socioeconomic status, facility type, tumor size, and surgical approach were associated with TO. Most of those variables are not modifiable and are associated with operative risks. Therefore, care should be taken before using TO to compare hospitals and surgeons, and case‐mix should be considered. In fact, our cohort was younger and had fewer comorbidities than those in previous reports, and we only included patients with early‐stage lung cancer and lobectomy, which might be one of the reasons for the higher TO in our study than in previous reports.

Because most of the individual criteria for TO are already known to be related to survival, such as complete resection,^20^ postoperative pulmonary complication,^28^ and readmission within 30 days,^29^ it is not surprising that the achievement of TO is associated with favorable OS. Previous reports of NCBD have shown an association of TO with improved survival, but have not shown disease‐specific outcomes, such as recurrence.^15^ In the present study, we investigated the effect of TO on both survival and recurrence. To minimize confounding factors, we used propensity‐matched analysis. We found a significant difference in OS in the unmatched and matched cohorts. However, we failed to find any difference in recurrence between the cohorts in the competing risk model. This finding suggests that the survival benefit from achieving TO would be associated with decreased noncancer‐related deaths in our cohort.

This study has several limitations. This was a single center retrospective study which included a small number of patients. The rate of TO achievement and cause of failure were quite different from those in previous reports from Western countries. We did not find a significant difference in recurrence according to the TO achievement. This might be a type 2 error due to the small number of patients and the high rate of sufficient LN dissection. A nationwide prospective study including a large number of patients is necessary to confirm the presence of internal variation in lung cancer surgery and the association between TO and recurrence.

In conclusion, TOs can be useful in assessing surgical quality, and the assessment of TO provides hospitals and surgeons the opportunity to review their practice for ideal postoperative outcomes. TO is associated with lower medical costs and favorable OS; thus, caregivers participating in lung cancer surgery and perioperative care should make an effort to improve the quality of care and achieve TO. Further nationwide studies are necessary to understand the quality variation and national characteristics of lung cancer surgery.

## CONFLICT OF INTEREST

The authors declare that they have no actual or potential conflicts of interest regarding the work reported herein.
